# Sub-phenotyping in critical care: a valuable strategy or methodologically fragile path?

**DOI:** 10.1186/s40635-025-00769-1

**Published:** 2025-06-05

**Authors:** Jim M. Smit, Annemijn H. Jonkman, Jesse H. Krijthe

**Affiliations:** 1 Data Science Group, Institute for Computing and Information Sciences, Radboud University, Nijmegen, The Netherlands; 2 Pattern Recognition & Bioinformatics Group, Delft University of Technology, Delft, The Netherlands; 3 Department of Intensive Care, Erasmus MC - University Medical Center Rotterdam, Rotterdam, The Netherlands

In her pioneering work, Calfee et al. [1] addressed the clinical and biological heterogeneity of acute respiratory distress syndrome (ARDS), a factor likely contributing to the poor track record of randomized trials (RCTs) in this patient population. Using latent class (or profile) analysis (LCA), a method for identifying unobserved subgroups from observed data, they identified two distinct ARDS sub-phenotypes (hypo- and hyperinflammatory), which showed association with clinical outcomes and, crucially, heterogeneity of treatment effect (HTE) [2], demonstrating different responses to higher vs. lower PEEP regimes.

Their study sparked a growing trend in critical care research: identifying sub-phenotypes via LCA or clustering methods, followed by examining HTE for specific interventions. Similarly, the recent work by Meza-Fuentes et al. [3] presented two ARDS sub-phenotypes, suggesting their potential for guiding individualized treatment. As sub-phenotyping has gained more traction in the ICU community than in other medical fields, we wonder: are we pioneering a valuable strategy for HTE analysis, or embarking on a methodologically fragile path?

Various alternative HTE analysis strategies exist. Traditional ‘one-variable-at-a-time’ subgroup analyses (e.g., comparing subgroups based on PaO_2_/FiO_2_ [4]) may suffer from limitations including low power and multiple comparisons. Furthermore, patients could belong to multiple overlapping subgroups that may experience treatment effects of varying size and direction. Predictive HTE approaches [2] aim to overcome some of these limitations, using multivariable models that enable analysing HTE across multiple patient characteristics simultaneously. Kent et al. [2] distinguishes two main approaches: *risk modelling* and *effect modelling*. Risk models use patient covariates and outcomes, stratifying patients by predicted risk, independent of treatment assignment. It may detect clinically meaningful HTE due to the risk-magnification phenomenon: homogeneous relative effects across patients lead to larger absolute benefits in those at higher baseline risk. Effect models, by contrast, incorporate treatment assignments during training, modelling treatment–covariate interactions to estimate individualized treatment effects. This direct modelling of treatment effect is theoretically ideal for HTE detection, but also prone to overfitting. Sub-phenotyping takes a different approach to find HTE: here multivariable models are trained *only* on patient covariates, assigning individuals to sub-phenotypes (Fig. 1). Although excluding both patient outcomes and treatment assignments during model training may reduce overfitting risk, it cannot directly model treatment effects like effect modelling, nor can it directly leverage risk-magnification like risk modelling. Instead, this approach assumes that observing grouping of patients based on covariates alone is an indicator for treatment effect heterogeneity, which may be incorrect. Hence, sub-phenotyping may be ‘underfit’ for identifying HTE, as these models cannot learn how patient characteristics are associated to outcomes or treatment effects.

**Fig. 1:**
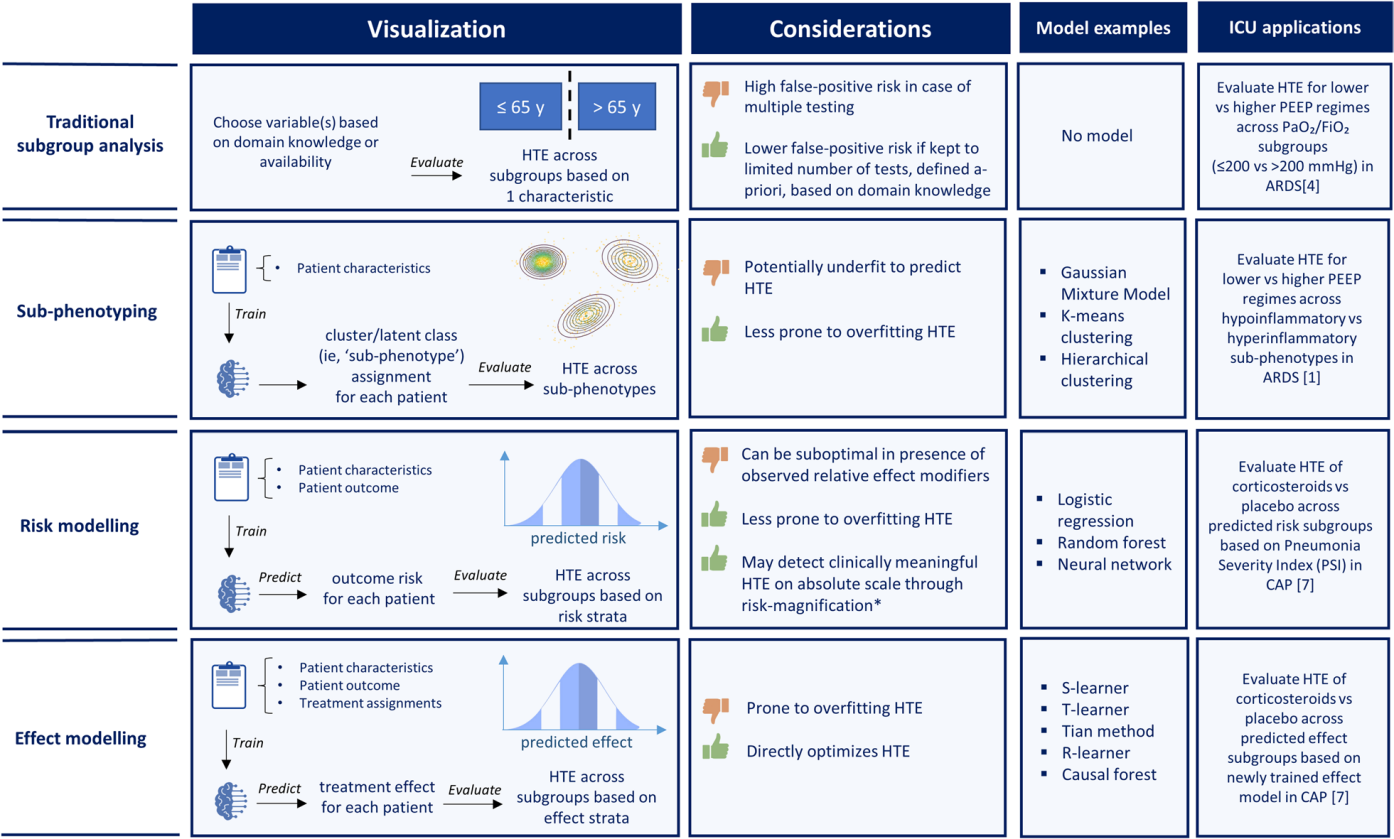
Schematic overview of approaches for HTE analysis, including traditional one-variable-at-a-time subgroup analysis, and multivariable HTE approaches (i.e., sub-phenotyping, risk modelling and effect modelling), with modelling considerations, and examples of used methodologies and applications in the ICU literature. *risk- (or benefit-) magnification: homogeneous relative effects across patients lead to larger absolute benefits in those at higher baseline risk

A recent position paper [5] reported strong consensus that certain ARDS sub-phenotypes may help enrich RCTs and guide personalized management, while calling for further validation. While we agree that further validation is crucial, we want to emphasize that it is the observed HTE across sub-phenotypes that requires validation. In the absence of sufficiently validated HTE, using sub-phenotypes for RCT enrichment (and particularly for personalizing treatment) lacks justification, as it may inadvertently exclude patients who could benefit. As more versions of sub-phenotyping models are developed, and HTE for various interventions is examined, the risk of false positives findings due to multiple testing increases, echoing the pitfalls of traditional subgroup analyses. For example, the HTE observed between hypo- and hyperinflammatory sub-phenotypes for high versus low PEEP in the ALVEOLI trial [1] was not replicated in the LOVS trial [6], suggesting that the finding which sparked the sub-phenotyping trend may have been a false positive. This underscores the critical need for rigorous validation of HTE findings, ideally across more than one independent RCT dataset, and, importantly, *after* pre-registration of both the model and evaluation protocol. [7]

In conclusion, subdividing heterogeneous ICU syndromes into sub-phenotypes seems compelling. However, if the aim is to further personalize treatment through HTE detection, we must critically assess whether sub-phenotyping is the preferred approach, especially when risk and effect modelling are also feasible. Regardless of the approach, we concur with Meza-Fuentes et al. [3] that validation of HTE findings using independent data is crucial before informing clinical practice.

## Data Availability

Not applicable.
